# Radical Complexity

**DOI:** 10.3390/e23121676

**Published:** 2021-12-14

**Authors:** Jean-Philippe Bouchaud

**Affiliations:** 1 Capital Fund Management, 75007 Paris, France; jean-philippe.bouchaud@cfm.fr; 2 Académie des Sciences, 75006 Paris, France

**Keywords:** financial markets, covariance matrices, copulas, high-frequency trading, market stability, agent-based models

## Abstract

This is an informal and sketchy review of five topical, somewhat unrelated subjects in quantitative finance and econophysics: (i) models of price changes; (ii) linear correlations and random matrix theory; (iii) non-linear dependence copulas; (iv) high-frequency trading and market stability; and finally—but perhaps most importantly—(v) “radical complexity” that prompts a scenario-based approach to macroeconomics heavily relying on Agent-Based Models. Some open questions and future research directions are outlined.

## 1. From Random Walks to Rough (Multifractal) Volatility

Since we will never really know *why* the prices of financial assets move, we should at least make a model of *how* they move. This was the motivation of Bachelier in 1900 [1] when he wrote in the introduction of their thesis that *contradictory opinions in regard to (price) fluctuations are so diverse that at the same instant buyers believe the market is rising and sellers that it is falling*. He went on to propose the first mathematical model of prices: the Brownian motion. He then built an option pricing theory that he compared to empirical data available to him, which already revealed, quite remarkably, what is now called the volatility smile! (Looking at his table on p. 30, one clearly see a smile that flattens with the maturity of the options, as routinely observed nowadays. As we now understand, this flattening comes from the slow convergence of returns towards Gaussian random variables as the time-lag increases, see, e.g., [2]).

After 120 years of improvements and refinements, we are closing in on a remarkably realistic model, which reproduces almost all known stylised facts of financial price series. However, are we there yet? As Benoît Mandelbrot once remarked: *In economics, there can never be a “theory of everything”. However, I believe each attempt comes closer to a proper understanding of how markets behave.* In order to close the gap and justify the modern mathematical apparatus that has slowly matured, we will need to understand the interactions between the behaviour of zillions of traders—each with their or her own investment style, trading frequency, risk limits, etc. and the price process itself. Interestingly, recent research strongly suggests that markets self organise in a subtle way, as to be poised at the border between stability and instability. This could be the missing link—or the holy grail—that researchers have been looking for.

For many years, the only modification to Bachelier’s proposal was to consider that log-prices, not prices themselves, are described by a Brownian motion. Apart from the fact that this modification prevents prices from becoming negative, none of the flaws of the Bachelier model were seriously tackled. Notwithstanding, the heyday of Brownian finance came when Black and Scholes published their famous 1973 paper, with the striking result that perfect delta-hedging is possible. However, this is because, in the Black–Scholes world, price jumps are absent and crashes impossible. This is, of course, a very problematic assumption, especially because the fat-tailed distribution of returns had been highlighted as soon as 1963 by Mandelbrot, who noted, in the same paper, that *large changes tend to be followed by large changes, of either sign, and small changes tend to be followed by small changes*, an effect now commonly referred to as “volatility clustering” and captured by the extended family of GARCH models.

It took the violent crash of October 1987, exacerbated by the massive impact of Black–Scholes’ delta-hedging, for new models to emerge. The Heston model, published in 1993, is among the most famous post-Black–Scholes models, encapsulating volatility clustering within a continuous time, Brownian motion formalism. However, similar to GARCH, the Heston model predicts that volatility fluctuations decay over a single time scale; in other words, periods of high or low volatility have a rather well defined duration. This is not compatible with market data: volatility fluctuations have no clear characteristic time scale; volatility bursts can last anything between a few hours and a few years.

Mandelbrot had been mulling about this for a long while and actually, in 1974, proposed a model to describe a very similar phenomenon in turbulent flows called “multifractality”. He adapted their theory in 1997 to describe currency exchange rates, before Bacry, Muzy and Delour formulated a more convincing version of the model in 2000, which they called the *Multifractal Random Walk* (MRW) [3]. With a single extra parameter (interpreted as a kind of volatility of volatility), the MRW satisfactorily captures many important empirical observations: fat-tailed distribution of returns and long-memory of volatility fluctuations. In 2014, Gatheral, Jaisson and Rosenbaum introduced their now famous “Rough Volatility” model [4], which can be seen as an extension of the MRW with an extra parameter allowing one to tune at will the roughness of volatility, while it is fixed in stone in the MRW model. Furthermore, indeed, empirical data suggest that volatility is slightly less rough than what the MRW posits. Technically, the Holder regularity of the volatility is H=0 in the MRW and found to be H≈0.1 when calibrated within the Rough Volatility specification.

The next episode of the long saga came in 2009 when Zumbach noticed a subtle, yet crucial aspect of empirical financial time series: they are not statistically invariant upon time reversal [5]. The past and future are not equivalent, whereas almost all models to that date, including the MRW, did not distinguish the past from future. More precisely, past price trends, whether up or down, lead to higher future volatility but not the other way round. In 2019, following some work by P. Blanc, J. Donier and myself [6], A. Dandapani, P. Jusselin and M. Rosenbaum proposed to describe financial time series with what they called a “Quadratic Rough Heston Model” [7], which is a synthesis of all the ideas reviewed above. It is probably the most realistic model of financial price series to date. In particular, it provides a natural solution to a long standing puzzle, namely the joint calibration of the volatility smile of the S&P 500 and VIX options, which had eluded quants for many years [8]. The missing ingredient was indeed the Zumbach effect [9].

Is this the end of the saga? From a purely engineering point of view, the latest version of the Rough Volatility model is probably hard to beat. However, the remaining challenge is to justify how this particular model emerges from the underlying flow of buy and sell trades that interacts with market makers and high-frequency traders. Parts of the story are already clear; in particular, as argued by Jaisson, Jusselin and Rosenbaum in a remarkable series of papers, the Rough Volatility model is intimately related to the proximity of an instability [10] (see also [11]) that justifies the rough, multi-timescale nature of volatility. However, what is the self-organising mechanism through which all markets appear to settle close to such a critical point? Could this scenario allow one to understand why financial time series all look so much alike; stocks, futures, commodities, exchange rates, etc., share very similar statistical features, in particular in the tails. Beyond being the denouement of a 120-year odyssey, we would be allowed to believe that the final model is not only a figment of our mathematical imagination, but a robust, trustworthy framework for risk management and derivative pricing. The next step will be to generalise these models in a multivariate setting, capturing the various channels through which price fluctuations propagate between different stocks and asset classes. The description of linear correlations is already a headache (see next section), but they are in fact not enough to capture the complexity of *non-linear* dependence in financial markets, which we discuss in Section 3.

## 2. Random Matrix Theory to the Rescue

Harry Markowitz famously quipped that diversification is the only free lunch in finance. This is nevertheless only true if correlations are known and stable over time. Markowitz’ optimal portfolio offers the best risk-reward tradeoff, for a given set of predictors, but requires the covariance matrix—of a potentially large pool of assets—to be known and representative of the future realised correlations. However, the empirical determination of large covariance matrices is fraught with difficulties and biases. Interestingly, the vibrant field of the “Random Matrix Theory” has provided original solutions to this big data problem and suggests droves of possible applications in econometrics, machine learning or other large dimensional models.

However, even for the simplest two-asset bond/equity allocation problem, the knowledge of the forward looking correlation has momentous consequences for most asset allocators in the planet. Will this correlation remain negative in the years to come, as it has been since late 1997, or will it jump back to positive territories? However, compared to volatility, our understanding of correlation dynamics is remarkably poor, and, surprisingly, the hedging instruments allowing one to mitigate the risk of bond/equity correlation swings are nowhere as liquid as the VIX itself.

Thus, there are two distinct problems in estimating correlation matrices. One is lack of data; the other one is time non-stationarity. Consider a pool of *N* assets, with *N* large. We have at our disposal *T* observations (say daily returns) for each of the *N* time series. The paradoxical situation is this: even though each individual off-diagonal covariance is accurately determined when *T* is large, the covariance matrix as a whole is strongly biased unless *T* is much larger than *N* itself. For large portfolios, with *N* of a few thousands, the number of days in the sample should be in the tens of thousands—say 50 years of data. This is simply absurd: Amazon and Tesla did not even exist 25 years ago. Maybe use 5-min returns then, increasing the number of data points by a factor 100? Yes, except that 5-min correlations are not necessarily representative of the risk of much lower frequency strategies, with other possible biases creeping in the resulting portfolios.

So in what sense are covariance matrices biased when *T* is not very large compared to *N*? The best way to describe such biases is in terms of eigenvalues. One finds that the smallest eigenvalues are way too small and the largest eigenvalues are too large. This results, in the Markowitz optimisation program, in a substantial over-allocation on a combination of assets that happened to have a small volatility in the past, with no guarantee that this will persist looking forward. The Markowitz construction can therefore lead to a considerable under-estimation of the realised risk in the next period.

Out-of-sample results are of course always worse than expected, but Random Matrix Theory (RMT) offers a guide to (partially) correct these biases when *N* is large. In fact, RMT gives an optimal, mathematically rigorous, recipe to tweak the value of the eigenvalues so that the resulting “cleaned” covariance matrix is as close as possible to the “true” (but unknown) one in the absence of any prior information on the direction of the eigenvectors. Such a result, first derived by Ledoit and Péché in 2011 [12], is already a classic and has been extended in many directions (see, e.g., [13,14]. Its operational implementation and the quality of out-of-sample predictions were extensively reviewed in [15,16,17]. The underlying mathematics, initially based on abstract “free probabilities”, are now in a ready-to-use format, very similar to Fourier transforms or Ito calculus (see [17] for an introductory account). One of the exciting and relatively unexplored directions is to add some financially motivated prior, such as industrial sectors or groups, to improve upon the default “agnostic” recipe.

Now the data problem is solved as best as possible, but the stationarity problem pops up. Correlations, similar to volatility, are not fixed in stone but evolve with time. Even the sign of correlations can suddenly flip, as was the case for the S&P500/Treasuries during the 1997 Asian crisis. After 30 years of correlations staunchly in positive territory (1965–1997), bonds and equities have been in a “flight-to-quality” mode (i.e., equities down and bonds up) ever since. More subtle, but significant, changes of correlations can also be observed between single stocks and/or between sectors in the stock market. For example, a downward move of the S&P500 leads to an increased average correlation between stocks. Here again, RMT provides powerful tools to describe the time evolution of the full covariance matrix [18,19].

As I discussed in the previous section, stochastic volatility models have made significant progress recently and, now, encode feedback loops that originate at the microstructural level, see also Section 4. Unfortunately, we are very far from having a similar theoretical handle to understand correlation fluctuations, although Matthieu Wyart and I had proposed a self-reflexive mechanism in 2007 to account for correlation jumps, such as the one that took place in 1997 [20]. Parallel to the development of descriptive and predictive models, the introduction of standardised instruments that hedge against such correlation jumps would clearly serve a purpose. This is especially true in the current environment [21] where inflation fears could trigger another inversion of the equity/bond correlation structure, which would be possibly devastating for many strategies that—implicitly or explicitly—rely on persistent negative correlations. Markowitz diversification free lunch can sometimes be poisonous!

## 3. My Kingdom for the Right Copula

As I just discussed, assessing linear correlations between financial assets is already hard enough. What about *non-linear* correlations then? If financial markets were kind enough to abide to Gaussian statistics, non-linear correlations would be entirely subsumed by linear ones. However, this is not the case: genuine non-linear correlations pervade the financial world and are quite relevant, both for the buy side and the sell side. For example, tail correlations in equity markets (i.e., stocks plummeting simultaneously) are notoriously higher than bulk correlations. Another apposite context is the Gamma-risk of large option portfolios, the management of which requires an adequate description of quadratic return correlations of the underlying assets.

In order to deal with non-linear correlations, mathematics has afforded us with a seemingly powerful tool—“copulas” [22]. Copulas are supposed to encapsulate all possible forms of multivariate dependence. However, in the zoo of all conceivable copulas, which one should one choose to faithfully represent financial data?

Following an unfortunate but typical pattern of mathematical finance, the introduction of copulas twenty years ago has been followed by a calibration spree, with academics and financial engineers alike frantically looking for copulas to best represent their pet multivariate problem. However, instead of first developing an intuitive understanding of the economic or financial mechanisms that suggest some particular dependence between assets and construct adequate copulas accordingly, the methodology has been to brute-force calibrate copulas straight out from statistics handbooks. The “best” copula is then decided from some quality-of-fit criterion, irrespective of whether the copula makes any intuitive sense at all.

This is reminiscent of local volatility models for option markets: although these models make no intuitive sense and cannot describe the actual dynamics of the underlying asset, it is versatile enough to allow the calibration of almost any option smile. Unfortunately, a blind calibration of some unwarranted model (even when the fit is perfect) is a recipe for disaster. If the underlying reality is not captured by the model, it will most likely derail in rough times—a particularly bad feature for risk management (recall the use of Gaussian copulas to price CDOs before the 2008 crisis). Another way to express this point is to use a Bayesian language: there are families of models for which the "prior" likelihood is clearly extremely small because no plausible scenarios for such models emerge from market mechanisms. Statistical tests are not enough—the art of modelling is precisely to recognise that not all models are equally likely.

The best way to foster intuition is to look at data before cobbling up a model and come up with a few robust “stylised facts” that you deem relevant and that your model should capture. In the case of copulas, one interesting stylised fact is the way the probability that two assets have returns simultaneously smaller than their respective medians depends on the linear correlation between the said two assets. Such a dependence exists clearly and persistently in stocks, and strikingly, it cannot be reproduced by most “out-of-a-book” copula families.

In particular, the popular class of so-called “elliptical” copulas is ruled out by such an observation. Elliptical copulas assume, in a nutshell, that there is a common volatility factor for all stocks: when the index becomes more or less volatile, all stocks follow suit. A moment of reflection reveals that this assumption is absurd since one expects that volatility patterns are at least industry-specific. However, this consideration also suggests a way to build copulas specially adapted to financial markets. In Ref. [23], R. Chicheportiche and I showed how to weld the standard factor model for returns with a factor model for volatilities. Perhaps surprisingly, the common volatility factor is not the market volatility, although it contributes to it. With a relatively parsimonious parameterisation, most multivariate “stylised facts” of stock returns can be reproduced, including the non-trivial joint-probability pattern alluded to above.

I have often ranted against the over-mathematisation of quant models, favouring theorems over intuition and convenient models over empirical data. The reliance on rigorous but misguided statistical tests is also plaguing the field. As an illustration related to the topic of copulas, let me consider the following question: is the univariate distribution of standardised stock returns *universal*, i.e., independent of the considered stock? In particular, is the famous “inverse-cubic law” [24,25] for the tail of the distribution indeed common to all stocks?

A standard procedure for rejecting such an hypothesis is the Kolmogorov–Smirnov (or Anderson–Darling) statistics. Furthermore, lo and behold, the hypothesis is strongly rejected. However, wait—the test is only valid if returns can be considered as independent, identically distributed random variables. Whereas returns are close to being uncorrelated, non-linear dependencies along the time axis are strong and long-ranged. Adapting the Kolmogorov–Smirnov test in the presence of long-ranged “self-copulas” is possible [26] and now leads to the conclusion that the universality hypothesis *cannot* be rejected by such a test. Intuitively, this is because the presence of long-range correlations in volatility drastically limit the *effective* size of the data set. We have much less independent data than we think.

Here again, thinking about the problem before blindly applying standard recipes is of paramount importance to get it right. Furthermore, of course, if the “inverse-cubic law” is indeed universal, as again recently advocated in [25], we should try to understand why. Despite many efforts in that direction, it is fair to say that there is no consensus on the underlying mechanism responsible for such a critical-like behaviour, see Section 1 and Section 4.

The finer we want to hone in on the subtleties of financial markets, the more we need to rely on making sense of empirical data and to remember what the great Richard Feynman used to say: *It does not matter how beautiful your theory is, it does not matter how smart you are. If it does not agree with experiment, it is wrong.*

## 4. High-Frequency Trading and Market Stability

In the midst of the first COVID lockdown, the 10th anniversary of the infamous 6 May 2010 “Flash Crash” went unnoticed. At the time, fingers were pointed at High-Frequency Trading (HFT), accused of both rigging the markets and destabilising them. Research has since then confirmed that HFT results in significantly lower bid-ask spread costs and, after correcting for technological glitches and bugs, does *not* increase the frequency of large price jumps. In fact, recent models explain why market liquidity is intrinsically unstable: managing the risk associated to market-making, whether by humans or by computers, unavoidably creates destabilising feedback loops. In order to make markets more resilient, research should focus on better market design and/or smart regulation that nip nascent instabilities in the bud.

Since orders to buy or to sell arrive at random times, financial markets are necessarily most of the time unbalanced. In such conditions, market-makers play a crucial role in allowing smooth trading and continuous prices. They act as liquidity buffers that absorb any temporary surplus of buy orders or sell orders. Their reward for providing such a service is the bid-ask spread—systematically buying a wee lower and selling a wee higher and pocketing the difference.

What is the fair value of the bid-ask spread? Well, it must at least compensate the cost of providing liquidity, which is *adverse selection*. Indeed, market-makers must post prices that can be picked up if deemed advantageous by traders with superior information. The classic Glosten–Milgrom model provides an elegant conceptual framework to rationalise the trade-off between adverse selection and bid-ask spread but fails to give a quantitative, operational answer (see, e.g., [27] for a recent discussion). In a 2008 study [28], we came up with a remarkably simple answer: the fair value of the bid-ask spread is equal to the ratio of the volatility to the square-root of the trade frequency. This simple rule of thumb has many interesting consequences.

First, it tells us that for a fixed level of volatility, increasing the trade frequency allows market-makers to reduce the spread and, hence, the trading costs for final investors. The logic is that trading smaller chunks more often reduces the risk of adverse selection. This explains in part the rise of HFT as modern market-making and the corresponding reduction in the spreads. Throughout the period 1900–1980, the spread on US stocks hovered around a whopping 60 basis points, whereas it is now only a few basis points [29]. In the meantime, volatility has always wandered around 40% per year—with of course troughs and occasional spikes, as we discuss below. In other words, investors were paying a much higher price for liquidity before HFT, in spite of wild claims that nowadays electronic markets are “rigged”. In fact, after a few prosperous years before 2010, high-frequency market-making has become extremely competitive and average spreads are now compressed to minimum values.

From this point of view, the economic rents available to liquidity providers have greatly decreased since the advent of HFT. However, has this made markets more stable, or has the decrease in the profitability of market-making also made them more fragile? The second consequence of our simple relation between spread and volatility relates to this important question. The point is that this relation can be understood in a two-way fashion: clearly, when volatility increases, the impact of adverse selection can be dire for market-makers who mechanically increase their spreads. Periods of high volatility can however be quite profitable for HFT since competition for liquidity providing is then less fierce.

However, in fact, higher spreads by themselves lead to higher volatility since transactions generate a larger price jump—or even a crash when liquidity is low and the order book is sparse. Thus, we diagnose a fundamental destabilising feedback loop, intrinsic to any market-making activity:

volatility⟶higherspreadsandlowerliquidity⟶morevolatility.

Such a feedback loop can actually be included in stochastic order book models (such as the now commonly used family of “Hawkes processes” [30]). As the strength of the feedback increases, one finds a phase transition between a stable market and a market prone to *spontaneous liquidity crises*, even in the absence of exogenous shocks or news [31].

This theoretical result suggests that when market-makers (humans or machines) react too strongly to unexpected events, liquidity can enter a death spiral. However, it is difficult to blame them since they are at risk of losing a full year of profit in a single adverse jump. As an old saying goes, *liquidity is a coward, it is never there when it is needed*. Liquidity can only be gossamer.

Such a paradigm allows one to understand why a large fraction of price jumps occur without any significant news—rather, they result from endogenous, unstable feedback loops [32,33]. Empirically, the frequency of 10-sigma daily moves of US stock prices has been fairly constant in the last 30 years, with no significant change between the pre-HFT epoch and more recent years [27]. Even the 6 May 2010 Flash Crash has a pre-HFT counterpart: on 28 May 1962, the stock market plunged 9% within a matter of minutes, for no particular cause, before recovering—much of the same weird price trajectory as in 2010. Our conjecture: markets are intrinsically unstable and have always been so. As noted in Section 1 above, this chronic instability may lie at the heart of the turbulent, multiscale nature of financial fluctuations and the universal power-law of the distribution of returns [24,25].

Can one engineer a smart solution that make markets less prone to such dislocations? From our arguments above, we know that the task would be to crush the volatility/liquidity feedback loop by promoting liquidity provision when it is on the verge of disappearing. One idea would be to introduce dynamical make/take fees, which would make cancellations more costly and limit order posting more profitable depending on the current state of the order book. These fees would then funnel into HFT’s optimisation algorithms and (hopefully) drive the system away from the regime of recurrent endogenous liquidity crisis.

## 5. Radical Complexity and Scenario Based Macro-Economics

Good science is often associated with accurate, testable predictions. Classical economics has tried to conform to this standard and developed an arsenal of methods to come up with precise numbers for next year’s GDP, inflation and exchange rates, among (many) other things. Few, however, will disagree with the fact that the economy is a complex system, with a large number of heterogeneous interacting units of different categories (firms, banks, households, public institutions) and very different sizes. In such complex systems, even qualitative predictions are hard. Thus, maybe we should abandon our pretense of exactitude and turn to another way to do science based on scenario identification. Aided by qualitative (agent based) simulations, swans that appear black to the myopic eye may in fact be perfectly white.

The main issue in economics is precisely about the emergent organisation, cooperation and coordination of a motley crowd of micro-units. Treating them as a unique representative firm or household risks throwing the baby out with the bathwater. Understanding and characterising such emergent properties is however difficult: genuine surprises can appear from micro- to macro-. One well-known example is the Schelling segregation model: even when all agents prefer to live is mixed neighbourhoods, myopic dynamics can lead to completely segregated ghettos [34]. In this case, Adam Smith’s invisible hand badly fails.

More generally, slightly different micro-rules/micro-parameters can lead to very different macro-states: this is the idea of “phase transitions”; sudden discontinuities (aka crises) can appear when a parameter is only slightly changed. Because of feedback loops of different signs, heterogeneities and non-linearities, these surprises are hard, if not impossible, to imagine or anticipate, even aided with the best mathematical apparatus.

This is what I would like to call “Radical Complexity”. Simple models can lead to unknowable behaviour, where “Black Swans” or “Unknown Unknowns” can be present, even if all the rules of the model are known in detail. In these models, even probabilities are hard to pin down, and rationality is de facto limited. For example, the probability of rare events can be exponentially sensitive to the model parameters and, hence, unknowable in practice [35]. In these circumstances, precise quantitative predictions are unreasonable. However, this does not imply the demise of the scientific method. For such situations, one should opt for a more qualitative, scenario-based approach, with emphasis on mechanisms, feedback loops, etc., rather than on precise but misleading numbers.

Establishing the list of possible (or plausible) scenarios is itself difficult. We need numerical simulations of Agent-Based Models (ABMs). While it is still cumbersome to experiment on large-scale human systems (although more and more possible using web-based protocols), experimenting with ABMs is easy and fun and indeed full of unexpected phenomena. These experiments in silico allow one to elicit scenarios that would be nearly impossible to imagine because of said feedback loops and non-linearities. Think, for example, of the spontaneous synchronisation of fireflies (or of neuron activity in our brains). It took nearly 70 years to come up with an explanation. Complex endogenous dynamics is pervasive but hard to guess without appropriate tools.

Experimenting with Agent-Based Models is interesting on many counts. One hugely important aspect is, in my opinion, that it allows to teach students in a playful, engaging way how complex social and economic systems work. Such simulations would foster their intuition and their imagination, much like lab experiments train the intuition of physicists about the real world beyond abstract mathematical formalism. Of course, similar to physics curriculums, experimenting with ABMs should be taught in parallel to, and not instead of, standard analytical models.

Creating one’s own world and seeing how it unfolds clearly has tremendous pedagogical merits. It is also an intellectual exercise of genuine value: if we are not able to make sense of an emergent phenomenon within a world in which we set all the rules, how can we expect to be successful in the real world? We have to train our minds to grasp these collective phenomena and to understand how and why some scenarios can materialise and others not. The versatility of ABMs allows one to include ingredients that are almost impossible to accommodate in standard economic models and explore their impact on the dynamics of the systems [36,37], in particular the inability of some of these a priori well-behaved economic models to ever reach equilibrium [38]. For a recent review of macroeconomic ABMs, see, e.g., [39].

ABMs are often spurned because they are generally hard to calibrate, and therefore, the numbers they spit out cannot and should not be taken at face value. (For an interesting discussion of why ABMs are not yet part of mainstream economics, see [40,41]). They should rather be regarded as all-purpose *scenario generators*, allowing one to shape one’s intuition about phenomena to uncover different possibilities and reduce the realm of Black Swans. The latter are often the result of our lack of imagination or of the simplicity of our models, rather than being inherently impossible to foresee.

Expanding the study of toy-models of economic complexity will create a useful corpus of scenario-based, qualitative macroeconomics [36,42,43], perhaps boosted by the recent Nobel prize of Giorgio Parisi. Instead of aiming for precise numerical predictions based on unrealistic assumptions, one should make sure that models rely on plausible causal mechanisms and encompass all plausible scenarios, even when these scenarios cannot be fully characterised mathematically. A qualitative approach to complexity economics should be high on the research agenda. As Keynes said, *it is better to be roughly right than exactly wrong.*
